# Integrated DNA-based/biochemical screening for early diagnosis of multiple endocrine neoplasia type 2A (MEN2A)

**DOI:** 10.7555/JBR.27.20120121

**Published:** 2013-02-20

**Authors:** Qin Cui, Wen Wang, Zhenzhen Fu, Xin Shao, Zhihong Zhang, Mei Zhang, Xianxia Ju, Kunlin Wang, Jiawei Chen, Hongwen Zhou

**Affiliations:** a Department of Geriatrics, Department of Cadres, Tong Ling People's Hospital, Tongling, Anhui 244000, China;; b Department of Endocrinology, Wuxi No.2 People's Hospital, Wuxi, Jiangsu 214002, China;; c Department of Endocrinology, the First Affiliated Hospital, Nanjing Medical University, Nanjing, Jiangsu 210029, China;; d Department of Endocrinology, Nanjing Municipal Chinese Traditional Medical Hospital, Nanjing, Jiangsu 210001, China;; e Department of Pathology, the First Affiliated Hospital, Nanjing Medical University, Nanjing, Jiangsu 210029, China

**Keywords:** MEN-2A, RET mutation, medullary thyroid carcinoma, Chinese, *RET* proto-oncogene, Calcitonin screening, DNA-based screening

## Abstract

Multiple endocrine neoplasia type 2A (MEN2A), a subtype of MEN2, is characterized by medullary thyroid cancer, pheochromocytoma, and primary hyperparathyroidism. A Han Chinese pedigree with MEN2A was investigated following confirmation of the proband's diagnosis by pathological findings and DNA/biochemical screening. DNA samples from 4 other family members were collected and exon 5, 8, 10, 11, 13, 16 and 18 of the *RET* proto-oncogene were sequenced and then analyzed. A missense mutation of TGG (Trp) to TGC (Cys) at codon 634 (the classic MEN2A mutation) in exon 11 of the *RET* gene was detected in 3 family members, including the proband. Sequencing data were compared with the human gene mutation database. Elevated serum calcitonin level was detected initially; medullary thyroid carcinoma was revealed in the 3 cases and adrenal pheochromocytoma was also found in the proband. Elective operations were successfully performed on the adrenal and thyroid glands because of pheochromocytoma and medullary thyroid carcinoma. Our case study confirms that integrated DNA-based/biochemical screening is crucial for early diagnosis of MEN2A and is helpful in the screening of their relatives. In addition, DNA-based screening may occasionally uncover a previously unknown *RET* sequence.

## INTRODUCTION

There are three distinct variants associated with multiple endocrine neoplasia type 2 (MEN2): (1) MEN2A, which is characterized by medullary thyroid carcinoma, pheochromocytoma and primary hyperparathyroidism; (2) MEN2B, which in addition to medullary thyroid carcinoma and pheochromocytoma, patients may develop marfanoid habitus, or intestinal and mucosal ganglioneuromatosis and familial medullary thyroid carcinoma[1]. In 1968, Steiner et al.[2] described the first MEN2A in a family with medullary thyroid carcinoma, pheochromocytoma and primary hyperparathyroidism. MEN2A is an autosomal dominate syndrome with an estimated prevalence of 1 per 40,000 in the United States[3]. However, there is still no data available for the prevalence of MEN2A in large Chinese populations. Other rare clinical syndromes related with MEN2A were also reported, such as cutaneous lichen amyloidosis and hirschsprung disease.

The MEN2A syndromes are caused by germline mutations in the *RET* proto-oncogene (OMIM 1171400), which is located on chromosome 10q11.2 and consists of 21 exons. It has been reported that 95% of MEN-2A patients are associated with mutations at codon 634, 620 and 618. Herein, we report 3 patients from the same family with a germ line *RET* mutation in codon 634, the classic MEN2A mutation. Our practice has shown that the combination of biochemical detecting (calcitonin and catecholamine) with DNA-based screening (*RET* proto-oncogene mutation analysis) can help identify individuals at risk of developing MEN2. There is good evidence to support that medullary thyroid carcinoma has not developed as long as the basal calcitonin levels remain within normal limits. Nearly one-third of pediatric *RET* gene carriers with increased basal calcitonin levels are believed to harbor occult medullary thyroid carcinoma[4]. This screening program makes it possible for individual decisions with regard to the timing of prophylactic thyroidectomy.

## SUBJECTS AND METHODS

### Subjects

**Fig. 1:**
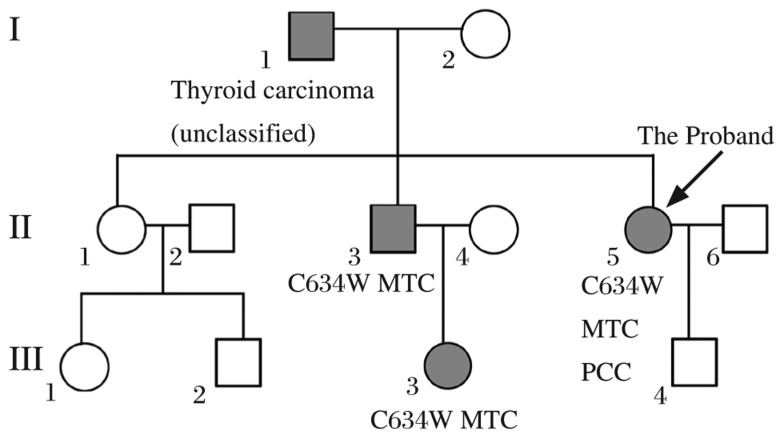
Pedigree of the family. Squares and circles indicate males and females, respectively. MTC: Medullary Thyroid carcinoma. PCC: Pheochromocytomas.

The proband (II-3), a 55-year-old woman who presented with a 2-year history of type 2 diabetes with weight loss, polydipsia and polyphagia, was hospitalized because of a 7-day history of dizziness, vomiting and asthenia. Biochemical examination and image scans showed a high serum level of calcitonin, urinary catecholamines, thyroid gland enlargement, and bilateral adrenal tumors. Her father (II-1) had a history of unclassified thyroid carcinoma, and her niece (III-3) was diagnosed with medullary thyroid carcinoma and treated in another hospital. Given the clinical data and the family history, the diagnosis of MEN2A was strongly recommended. We then studied the pedigree of this patient (***Fig 1***). Ten normal, unrelated subjects without a familial history of medullary thyroid carcinoma were collected as controls. All subjects gave informed written consent.

### Clinical investigation

Clinical evaluation of MEN2A was performed according to the published criteria[1]. The clinical and diagnostic data of the family constellation, including age, gender and physical examination were collected. To obtain further clinical features, we evaluated the biochemical profiles of the 5 family members of the proband (II-3, III-3 and III-4). The following parameters were studied: serum calcitonin, parathyroid hormone, thyroid hormones, serum calcium, and serum phosphorus. Further screening for medullary thyroid carcinoma was done by Doppler ultrasound (US) and fine needle aspiration cytology. US was also used for screening of pheochromocytoma and primary hyperparathyroidism. Treatment of medullary thyroid carcinoma and pheochromocytoma was accomplished by surgery and subsequent follow-up was carried out.

### Histopathology

The surgical specimens of the thyroid gland and adrenal gland were studied routinely. Medullary thyroid carcinoma and pheochromocytoma were established by the conventional H&E staining. Serial sections were examined for calcitonin, thyroglobulin, CK19, thyroid transcription factor 1, synaptophysin, and chromogranin A protein staining using an immunohistochemical method, in order to exclude other sources of thyroid cancers.

### *RET* mutation analysis

Genomic DNA was extracted from 5 family members of the proband (II-1, 2, 3, III-3, III-4) and 10 normal subjects. DNA extraction, using 200 µL of whole blood was done following the manufacturer's procedure (Invitrogen Carlsbad, CA, USA). DNA was then kept at -20°C. Exons 5, 8, 10, 11, 13, 16 and 18) of the *RET* gene were amplified using polymerase chain reaction (PCR). The annealing temperature and primers are listed in ***Table 1***. The PCR reactions were performed in a final volume of 20 µL containing 80 ng DNA, 0.5 µmol/L of each primer, and 10 µL PCR buffer containing 1.5 mmol/L MgCl_2_, 200 µmol/L dNTPs, 2.5 U *Taq* DNA polymerase. The amplification was performed in a thermal cycler, (Bio-Rad PTC-200 Peltier Thermal Cycler, Hercules, CA, USA) with preheating at 95°C for 5 min, followed by 30 cycles at 94°C for 30 seconds, annealing at 55-64°C for 45 minutes and extension at 72°C for 45 seconds, and a final extension at 72°C for 10 minutes. The PCR products were stored at -20°C until the following purification and direct sequencing by Invitrogen.

**Table 1:** Primers and annealing temperature used for *RET* sequencing

Exon	Forward primer(5′-3′)	Reverse prime(5′-3′)	Annealing temp(°C)	Product size(bp)
5	gacgtgcagcattctaaggt	catgaagagcgagcacctca	60	310
8	ttgggcactagctggacg	accttcccaagtccagagt	62	385
10	cagaaaggcactgtgaccaa	gggacctcagatgtgctgtt	55	420
11	ccatgaggcagagcatacg	cctcctctgcccagcgttg	60	421
13	agcctcaagcagcatcgt	gcagtagggaaagggagaaag	64	341
14	tggctcctggaagaccca	gtgggctagagtgtggca	58	350
15	ggcctgacgactcgtgct	caaagaatgacgatcctgctaat	64	546
16	agggatagggcctgggcttc	taacctccaccccaagagag	59	192
18	gctgtccttctgagacctg	cacactgggaactctgagg	58	227

## RESULTS

### Clinical data of the proband

**Fig. 2:**
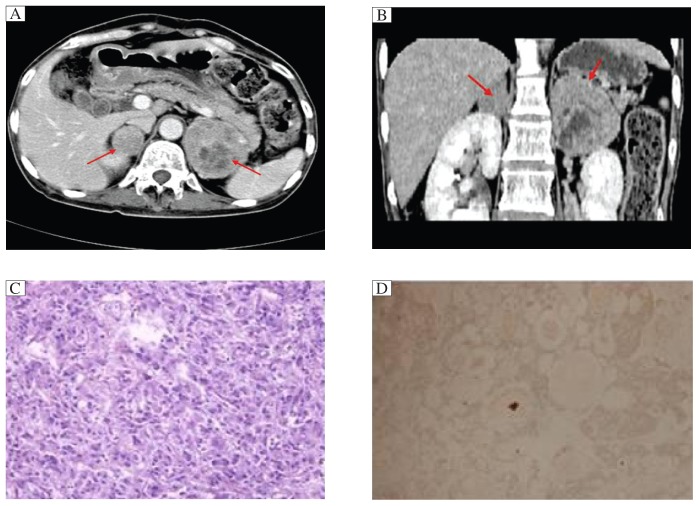
Results of CT and histopathological examination. A: Coronal CT image. B: Sagittal CT image. The red arrows indicate pheochromcytoma. C: H&E staining (40×) of pheochromcytoma. D: Immunohistochemical staining of thyroid sections: calcitonin (+).

Physical examination on the proband revealed hypertension (140/100 mmHg), bilateral thyroid nodule and a 5×5 cm lump in the left upper quadrant. Endocrinological testing revealed elevated levels of calcitonin and urinary catecholamines (***Table 2***). Computerized tomography disclosed a large left adrenal mass (6.0×6.2×5.0 cm), which was confirmed as phaeochromcytoma by histopathological examination during and after surgery. US disclosed bilateral thyroid nodule containing calcifications. The patient underwent total right thyroidectomy and 70% left thyroidectomy. Histopathology of the surgical specimens of the thyroid gland was positive for medullary thyroid carcinoma. Immunohistochemical staining displayed calcitonin (+), TG(-), TTF1(-), CgA(-), CK19(-) and Syn(-). Calcitonin was 26.1 pg/mL 2 months after surgery. The results of CT scanning, H&E staining and immunohistochemistry are shown in ***Fig 2***.

**Table 2:** Biochemical findings for the proband (II 5)

Item	Proband	Reference values
24-hour urinary epinephrine (ug)	983.45	< 20
24 hour urinary norepinephrine (ug)	290.60	< 90
Serum calcium (mmol/L)	2.47	2.15-2.65
Serum phosphate (mmol/L)	1.33	0.86-1.86
Calcitonin (pg/mL)	1453.00	0-100
Parathyroid hormone (pg/mL)	71.90	10-88

### Localization of the mutation

**Fig. 3:**
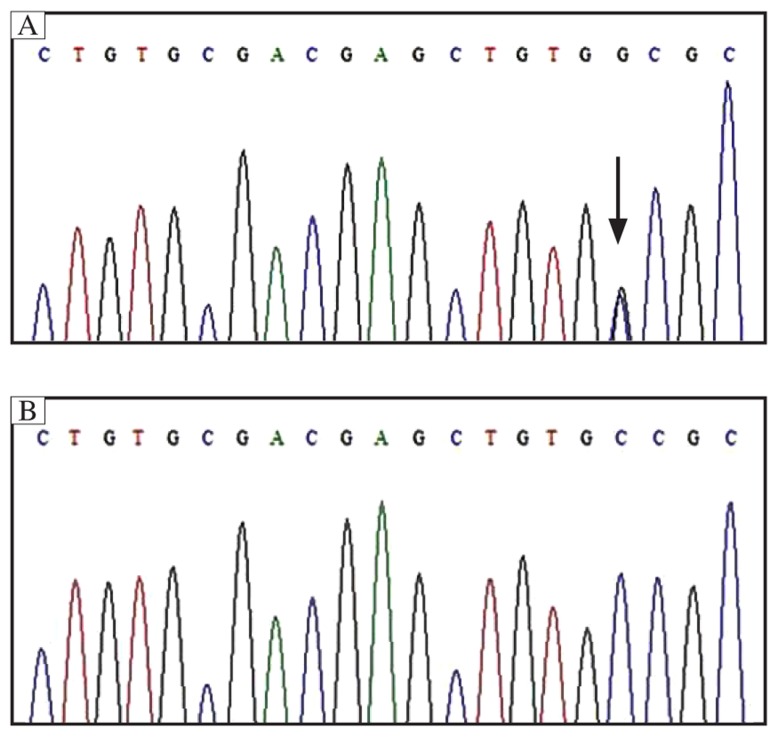
Sequencing of exon 11 of the *RET* proto-oncogene. A: The proband case, and the mutation (C634W, TGG-to-TGC) is marked by an arrow. B: The normal control is shown as comparison.

DNA sequence analysis for 9 exons of the *RET* gene listed in ***Table 1*** revealed a transversion of TGC-to-TGG at codon 634 of exon 11 (***Fig 3***). We then studied exon I1 on the four family members (II-1, II-2, III-3, III-4). We found the same heterozygote mutation of exon 11 in II-3 and III-3. No mutation of exon I-1 was found in II-1 and III-4.

### Management based on genetic information

The parents of the index patient died a few years ago, so DNA information about them is unavailable. However the father of the proband (I-1) was diagnosed with unclassified thyroid carcinoma. The niece of the proband (III-3) had a definite history of medullary thyroid carcinoma and was operated on, prior to our study, at the age of 22 years, but she had not been screened for gene mutations until our present study. Based on our *RET* mutation analysis, physical examination, biochemical evaluation and B ultrasonography were performed on the three family members for MEN-2A screening (***Table 3***). Consistent with the mutation analysis, elevated calcitonin value and bilateral thyroid nodules containing calcification were revealed on the brother of the proband (II-2). Total thyroidectomy was performed the same as the proband. Basal calcitonin level was nearly normal at 2 months post surgery.

**Table 3:** Examination findings of the 4 relatives of the proband

Relatives	Age (years)	BP (mmHg)	Calcitonin (pg/mL) Basic, post operation	PTH (pg/mL)	B Ultrasonography Thyroid Adrenal Parathroid
II-1	55	140/80	29.7, N	24.90	(-) (-) (-)
II-2	51	120/90	138, 130	49.40	(++) (-) (-)
III-3	26	100/60	N, 30.46	49.50	(+) (-) (-)
III-4	24	90/60	43.3, N	40.30	(-) (-) (-)

BP: blood pressure; N: No data available; PTH: parathyroid hormone. (-): No neoplasm was detected. (+): Neoplasm was detected in bilateral glands. (++): bilateral thyroid nodule associated with calcification.

## DISCUSSION

The proband from this family a 55-year-old woman (II-2), characterized with higher serum level of calcitonin, higher urinary catecholamines and bilateral adrenal tumors. The diagnosis of medullary thyroid carcinoma and pheochromocytoma was given and confirmed by histopathology of the surgical specimens. Her father (II-1) had a history of unclassified thyroid carcinoma, and her niece (III-3) was diagnosed with medullary thyroid carcinoma and was treated in another hospital. Given the clinical data and the family history, the diagnosis of MEN2A was strongly recommended. DNA-based screening (*RET* proto-oncogene mutation analysis) combined with biochemical screening (calcitonin and catecholamine) were applied in this family. *RET* proto-oncogene mutation analysis disclosed a missense mutation of TGC-to-TGG at codon 634 of exon 11 among three family members in this case study.

There are various clinical manifestations in MEN2A patients and they may differ among members of the same family. The patient may be asymptomatic and then exhibits symptoms of hyperfunctioning of different endocrine glands, one after the other after long intervals, or two endocrine glands simultaneously. The thyroid is usually the first gland to be affected and most often develops medullary thyroid carcinoma (nearly 100%) with multifocal bilateral localization. The risk for developing pheochromocytoma and primary hyperparathyroidism is 50% and 10-30%, respectively[5]. However, the incidence varies in different kins. In this case report, only the proband presented typical symptoms with increased catecholamine levels including paroxysmal hypertension, headaches and palpitations and thyroid endocrinological testing revealed a significantly higher calcitonin level. The other 3 patients had no clinical manifestations of pheochromocytoma except for medullary thyroid carcinoma. There was no evidence of primary hyperparathyroidism in the family.

The diagnosis of MEN2A largely depends on clinical manifestations, laboratory tests and imaging. Serum calcitonin is a tumor marker of medullary thyroid carcinoma with high sensitivity and specificity, and levels increase notably in the disease. The determination of serum calcitonin is of great importance for monitoring metastasis or tumor recurrence. Excluding two patients (I-1, III-4, no available data), the other 2 patients (II-2, II-5) had abnormally increased calcitonin levels, which is consistent with features of MEN2A. The detection of epinephrine and norepinephrine in plasma or 24 hour urine can contribute to the diagnosis of pheochromocytoma[6]. Serum calcium, phosphate and parathyroid hormone levels can be used to diagnose primary hyperparathyroidism considering that there are usually no specific clinical manifestations in parathyroid hyperplasia patients. Imaging screening studies including B ultrasonography, computer tomography, magnetic resonance imaging (MRI), and radioisotope scanning, are mainly used to identify the location and size of medullary thyroid carcinoma, pheochromocytoma and primary hyperparathyroidism. However, genetic screening is the most reliable screening and diagnostic method. In recent years, with the application of *RET* gene examination, different *RET* mutations have been well-studied[7],[8]. The advantage of genetic analysis is that it can help identify young carriers at an earlier stage of disease with highly reliable accuracy and stability in the absence of any other biochemical and pathological changes. In addition, it is effective in the differential diagnosis of MEN2A, MEN2B, familial and sporadic medullary thyroid carcinoma[9]. Nowadays, many mutational sites have been reported with 95% of them locating at codon 634 of exon 11 and codon 609/611/618/620 of exon 10[10],[11]. When an index case is identified, all family members at risk should undergo testing, regardless of their age. In families where the mutational site is already known, *RET* sequencing can be limited to the site of the known mutation[12]. In the present study, we focused on the hot-spot *RET* exons (10, 11, 13, 14, 15 and 16) and other 3 *RET* regions with a lower incidence rate (5, 8 and 18)[8]–[10],[13]. After DNA analysis, we found a missense mutation of TGC-to-TGG at codon 634 of exon 11. As a result, we sequenced exon 11 of the other family members. The parents of the proband died prior to our study; therefore, we were unable to obtain any DNA information from them. Because the father (I-1) had a definite thyroid carcinoma history, there was a great possibility that these MEN2A patients got the mutation from the father's side (I-1) In our clinical practice, we disclosed a MEN2A patient ((II-2) without any symptoms by integrated DNA-based screening (*RET* proto-oncogene mutation analysis) / biochemical screening (calcitonin and catecholamine). It is important to apply this kind of screening program to determine the subjects who are at risk of suffering from MEN2A. The DNA information could be of great importance for further studies in appropriate families.

Surgery is the definitive treatment for MEN2A and the operation for pheochromocytoma should be performed before thyroidectomy for medullary thyroid carcinoma. Preventive thyroidectomy on a MEN2A gene mutation carrier can improve the cure rate to nearly 100%[14],[15]. Even though serum calcitonin level is within the normal range, young patients with probable MEN2A should undergo thyroidectomy[16]. Recently, the American Thyroid Association (ATA) has classified the risk level according to all known mutations and recognized the more aggressive nature of codon 634 mutations[17]. Prophylactic surgery on patients who had condon 634 mutations was recommended before 5 years of age[9]. The postoperative management of patients with MEN2A is also very important. Life-long hormone replacement is essential. In this case, thyroid hormone replacement and/or adrenocortical hormone replacement were given to patients after surgery, with regular endocrinological testing.

In conclusion, sequencing of the *RET* gene to detect germline mutations is now the standard screening test for MEN2 syndromes[2]. As clinicians, we should give great attention to patients with involvement of multiple endocrine glands, elevated hormone levels and their family members. Further examinations, including biochemical tests and imaging modalities, can be chosen depending on the utility, cost and availability. To date the youngest reported child with codon 634 mutation was one year of age[18]. In future studies, we should aim for deeper insights into the RET-signaling pathway and the translation of biochemical knowledge into treatment options for patients.
